# The Impact of Parental Media Attitudes and Mediation Behaviors on Young Children’s Problematic Media Use in China: An Actor–Partner Interdependence Mediation Model Analysis

**DOI:** 10.3390/bs15081141

**Published:** 2025-08-21

**Authors:** Chaopai Lin, Ying Cui, Xiaohui Wang, Xiaoqi Su, Limin Zhang, Qian Peng

**Affiliations:** 1 Department of Early Childhood Education, School of Education, Central China Normal University, Wuhan 430079, China; linchaopai@gmail.com; 2 Department of Early Childhood Education, School of Education, Guangzhou University, Guangzhou 510006, China; cuiying@e.gzhu.edu.cn (Y.C.); wangxiaohui@e.gzhu.edu.cn (X.W.); 3 School of Education, Guangzhou University, Guangzhou 510006, China; suxiaoqi@e.gzhu.edu.cn; 4 Department of Early Childhood Education, Faculty of Educational Sciences, South China Normal University, Guangzhou 510631, China

**Keywords:** actor–partner interdependence model, parents’ attitudes toward screen use, parental mediation behaviors, problematic media use

## Abstract

Young children’s problematic media use (PMU) is a growing concern, and parents are critical in shaping early digital habits. However, research often overlooks the dyadic interplay between mothers’ and fathers’ attitudes and parenting practices. This study examined how parents’ favorable attitudes toward child screen media (PASU) predict their own (actor) and their partner’s (partner) mediation behaviors, and how these behaviors subsequently mediate the path to children’s PMU. Drawing on survey data from 1802 matched urban Chinese mother–father pairs, we employed an Actor–Partner Interdependence Mediation Model (APIMeM) within a structural equation modeling (SEM) framework. This dyadic model simultaneously tested actor, partner, and indirect mediation paths connecting parental attitudes to PMU via eight specific parenting practices. Results showed that more positive PASUs predicted each parent’s own supportive behaviors (e.g., high-quality dialogue, autonomy support) but not restrictive limits. Partner effects were modest and asymmetric: mothers’ positive attitudes predicted greater knowledge in fathers, whereas fathers’ positive attitudes were linked to lower communication quality from mothers. Of all parenting dimensions, only higher communication quality (both parents) and mothers’ hands-on monitoring directly predicted lower PMU. Mediation analyses confirmed communication quality as the sole reliable pathway: each parent’s favorable attitudes indirectly lowered PMU by enhancing their own dialogue, but fathers’ attitudes simultaneously increased PMU by eroding mothers’ dialogue. These findings spotlight constructive conversation and coordinated dyadic strategies—especially safeguarding maternal dialogue—as critical targets for interventions aimed at curbing early PMU.

## 1. Introduction

Digital media is becoming increasingly prevalent in the lives of young children, many of whom start interacting with screens from an early age and frequently exceed recommended usage limits (Bergmann et al., 2022). Such early and prolonged exposure has led to growing concerns about problematic media use (PMU), defined not simply by the duration of media consumption but by excessive use that disrupts critical developmental domains (Domoff et al., 2019; Gentile et al., 2017; Swit et al., 2023). PMU in early childhood is linked to numerous negative outcomes, including impaired social–emotional skills, cognitive and attentional problems, sleep disturbances, and strained familial relationships (Gentile et al., 2017; McDaniel & Radesky, 2018; Swit et al., 2023).

These concerns are particularly relevant in the Chinese context, where digital media access among young children is notably widespread. National data indicate that 67.6% of Chinese preschoolers exceed one hour of screen time on weekdays (averaging 1.3 h), while weekend usage often surpasses two hours (averaging 2.6 h) (Hua et al., 2022). Moreover, regional studies report high proportions—such as 43.8% in a recent study—of preschoolers surpassing recommended screen time limits (Wang et al., 2022). This phenomenon occurs against the backdrop of high national internet penetration rates and significant cultural emphasis on academic achievement, creating distinct pressures and parenting challenges regarding media use within Chinese families (Lim & Wang, 2024).

The home environment serves as the primary context for young children’s digital media exposure, with parents playing a pivotal role as key architects of children’s domestic media ecology (Choy et al., 2024). Empirical evidence suggests that the preschool years constitute a critical period for establishing media use habits, wherein parents function as essential “gatekeepers” whose mediation practices significantly shape children’s media engagement patterns (Livingstone & Helsper, 2008). However, modifying parental mediation behaviors presents considerable challenges, as such changes require both habitual adjustments and long-term implementation of revised parenting strategies. Current research indicates that parental mediation approaches are fundamentally influenced by caregivers’ underlying media attitudes (Nikken & Schols, 2015). Consequently, this study focuses on transforming parental media attitudes as a strategic leverage point for optimizing mediation practices, thereby facilitating healthier media use habits in early childhood.

### 1.1. Theoretical Framework

The theoretical framework of this study integrates three complementary theoretical perspectives to systematically explore how intra-family dynamics influence preschoolers’ problematic media use (PMU).

First, Family Systems Theory serves as the macro-level guiding paradigm (Bowen, 1993). The theory suggests that the family is an interconnected emotional unit in which the behaviors, thoughts, and feelings of each member influence the others. Crucially, due to differences in roles, attitudes, and behaviors, mothers and fathers form distinct mother–child and father–child relationship subsystems. Therefore, to fully understand children’s problematic media use, parents must be examined as an interacting “duality,” with particular attention paid to the different roles that different family members may play and the interactions between them.

Second, within the broader context of the family system, Interactional Theory of Childhood Problematic Media Use (IT-CPU) identifies key proximal factors influencing children’s media use. The theory points out that parental attitudes toward media use and media-related parenting behaviors serve as proximal factors that directly influence children’s media engagement, with risk elements potentially leading directly to early problematic media use behaviors (Domoff et al., 2020). However, existing theories still fall short of explaining ‘what factors shape these important parenting behaviors.’ Therefore, this study aims to explore the driving factors behind parental media parenting behaviors and emphasize that this behavior is a key factor influencing children’s PMU.

Third, to explore the driving factors behind parental media parenting behaviors, this study introduced the Theory of Planned Behavior (TPB; Ajzen, 1991; Fishbein & Ajzen, 2011). This theory believes that an individual’s attitude is a key predictor of their own behaviors. This theory provides a theoretical basis for the expected ‘actor effect,’ namely that parents’ attitudes toward the media directly influence their own regulatory behaviors, with parents with positive attitudes more likely to implement supportive parenting behaviors (e.g., co-use, positive guidance), while parents with negative attitudes are more likely to adopt restrictive behaviors (Ajzen, 1991). Therefore, this theory clarifies the psychological mechanisms between the parental attitudes of individual parents and their mediation behaviors.

This integrated framework, which embeds IT-CPU and TPB within Family Systems Theory, not only enables us to systematically explore how parental attitudes translate into specific mediation behaviors but also reveals how these processes unfold within the complex interdependent dynamics of the entire family system, including the unique roles and mutual influences of mothers and fathers. This provides a more cohesive and nuanced perspective for understanding the factors influencing children’s problematic media use, addressing the limitations of previous research that insufficiently addressed the complex interactions within the parental dyad.

### 1.2. Parenting Behaviors and Children’s Media Use

Children are increasingly growing up in homes with screen media technologies, the Interactional Theory of Childhood Problematic Media Use (IT-CPU) suggests that parenting practices, specifically media-related parenting or parental mediation behaviors (PMB) as important proximal risk factors impacting PMU, with risk elements potentially leading directly to early problematic media use behaviors (Domoff et al., 2020; Livingstone & Helsper, 2008). Broadly defined, parental mediation encompasses the strategies parents employ to regulate, manage, or engage with their child’s media consumption. Classical research has identified three primary parental mediation strategies: restrictive mediation (setting limits on media content or duration), active mediation (discussing and explaining media content with children), and co-use or co-viewing (jointly engaging with media content alongside children) (Clark, 2011; Livingstone & Helsper, 2008; Nathanson, 2001; Valkenburg et al., 1999).

However, the complexities of contemporary digital environments necessitate refining these broad categories further (Nikken & Jansz, 2014; Peng et al., 2024). And existing research indicates that different mediation strategies have significantly varying impacts on children’s problematic media use. For instance, restrictive mediation (e.g., setting rules) can help children manage their time effectively and reduce issues of excessive media use (Vandewater et al., 2005). Active mediation (e.g., open communication) may enhance children’s media literacy, making them aware of the harms of excessive use and thereby reducing addiction tendencies (E. J. Lee & Kim, 2018; Liu et al., 2023). Furthermore, co-use was once considered a potentially positive intervention approach. However, studies point out that due to the portability and individualized usage characteristics of digital devices (e.g., smartphones, tablets), pure co-use (e.g., co-viewing) may be difficult to implement effectively (E. J. Lee & Kim, 2018). Parental monitoring and knowledge (e.g., understanding children’s online activities) have been proven to significantly reduce children’s media use time, thereby lowering the risk of problematic use (Marciano et al., 2022; Su et al., 2018). However, Mitchell et al. (2003) found that restrictive mediation (e.g., software controls) was more effective than parental monitoring in reducing children’s media use risks. Notably, regarding autonomy support, compared to adolescents, young children’s self-control abilities remain underdeveloped. Their problematic media use behaviors are more likely to be reinforced by the immediate gratification provided by media (Chang et al., 2019). This implies that broad classifications of mediation behaviors may obscure important nuances in parent-child media interactions. Therefore, in the current study, we employed a comprehensive, eight-domain measure—assessing communication frequency, communication quality, hands-on monitoring, sequence control, acquaintance monitoring, autonomy support, co-use, and parental knowledge—specifically designed to capture these complexities among Chinese parents of preschool-aged children (Zhou et al., 2022).

As such, this study intends to break through the previous general analysis of the impact of parental media parenting behaviors on young children’s problematic media use. Based on the established protective function of parental involvement, we hypothesized that active mediation (including communication frequency and communication quality), restrictive mediation (including hands-on monitoring, sequence control, acquaintance monitoring), co-use, and parental knowledge would negatively predict children’s problematic media use, but autonomy support would positively predict children’s problematic media use (Hypothesis 1).

### 1.3. The Role of Parental Attitudes Toward Media Use

While parenting behaviors are recognized as proximal influences on children’s media use, these behaviors themselves are shaped by parents’ underlying attitudes, beliefs, and perceptions regarding the role and impact of media. Parental attitudes toward screen use (PASUs)—reflecting parents’ beliefs about the benefits and harms of media, their value judgments on different types of content, and their overall stance on screen technologies in their children’s lives—serve as critical cognitive precursors influencing parental mediation efforts. Prominent theoretical frameworks, including the Theory of Planned Behavior (TPB; Ajzen, 1991; Fishbein & Ajzen, 2011), assert that attitudes toward specific behaviors significantly determine individuals’ intentions and subsequent behaviors. Therefore, parents’ attitudes toward the media directly influence their own regulatory behaviors. Similarly, cognitive consistency theories propose that individuals inherently seek alignment between their attitudes and behaviors (Festinger, 1957; Gawronski & Strack, 2012). Consistent with these theoretical perspectives, prior empirical research has repeatedly demonstrated that parents’ personal attitudes toward media significantly predict their choice of mediation strategies (Livingstone & Helsper, 2008; Nikken & Jansz, 2014; Radesky et al., 2014). For example, parents with a positive attitude toward media are more likely to implement active mediation and co-use (Nikken & Jansz, 2014; Nikken & Schols, 2015), and grant their children more autonomy to foster a positive family environment (H. E. Lee et al., 2022). By contrast, parents with a negative attitude are more inclined to adopt frequent monitoring (Cohen et al., 2023), establish restrictive media usage rules (Nikken & Schols, 2015), and actively discuss media content with their children (Nikken & Jansz, 2014). Thus, we hypothesized that parents’ more positive attitudes toward screen use would negatively predict their own restrictive mediation but positively predict their active mediation, co-use, autonomy support, and parental knowledge (actor effects) (Hypothesis 2a).

While the importance of parental mediation behaviors is widely acknowledged, research in this area has predominantly emphasized mothers’ roles, frequently over-looking the significant and potentially distinct contributions of fathers (Lamb, 2010; Wang et al., 2022). Fathers’ involvement is important, as their influence on children’s media use may differ significantly from mothers. For example, a study with Chinese preschoolers found that fathers’ own screen time independently predicted their children’s screen usage, distinct from maternal influences (Wang et al., 2022). From the perspective of Family Systems Theory, the family is an interconnected emotional unit. However, due to differences in roles, attitudes, and behaviors, mothers and fathers form distinct mother–child and father–child relationship subsystems. Therefore, both parents contribute uniquely to creating the family environment that shapes child outcomes (Bowen, 1993; Cox & Paley, 2003). Studies focusing exclusively on mothers thus fail to capture the complexity of the entire family system and may overlook critical coparenting dynamics, such as how parental coordination—or a lack thereof—in managing media impacts the child’s experiences and the effectiveness of mediation strategies (Feinberg, 2003; McDaniel & Radesky, 2018). To address this gap and provide a more comprehensive understanding, the current study extends beyond single-parent analyses by investigating how both maternal and paternal media-related parenting behaviors are associated with problematic media use among young Chinese children.

To rigorously capture these dyadic interactions, particularly how parents’ attitudes influence their own and their partners’ mediation behaviors, the current study employs the Actor–Partner Interdependence Model (APIM; Kenny et al., 2020). This model explicitly models the interdependence between dyad members, enabling a nuanced understanding of individual and cross-partner influences of parental attitudes on mediation behaviors within couples. Based on these arguments, we hypothesized that parents’ more positive attitudes toward screen use would also negatively predict their partner’s restrictive mediation but positively predict their partner’s active mediation, co-use, autonomy support, and parental knowledge (partner effects) (Hypothesis 2b).

By integrating these theoretical considerations, this not only enables us to systematically explore how parental attitudes translate into specific mediation behaviors but also reveals how these processes unfold within the complex interdependent dynamics of the entire family system, including the unique roles and mutual influences of mothers and fathers. Integrating these pathways, we hypothesized that parents’ more positive attitudes toward screen use would positively influence children’s PMU via their impact on restrictive mediation but negatively influence children’s PMU via their impact on active mediation, co-use, autonomy support, and parental knowledge, considering both actor and partner mediation pathways within the model (Hypothesis 3).

### 1.4. The Current Study

**Figure 1:**
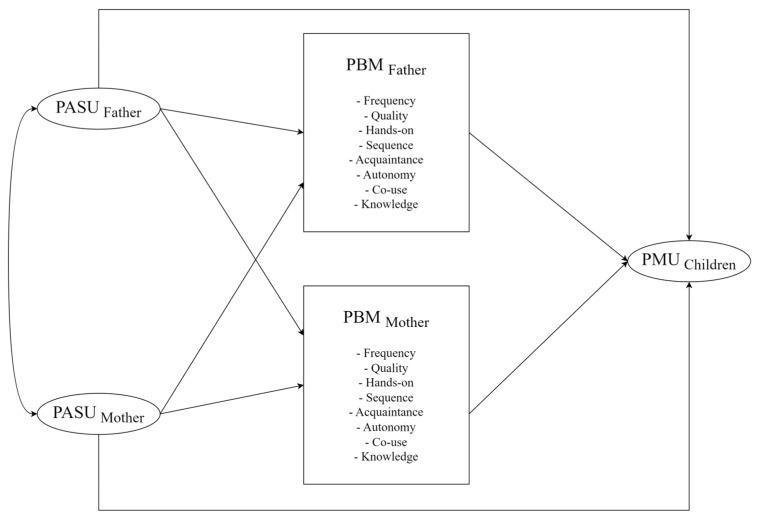
The theoretical hypothesis model of this study. Note: In the statistical model, parallel mediation analysis was conducted where each of the listed PMB dimensions was tested simultaneously as an independent mediator linking PASU to the PMU. For clarity, only the main paths are shown.

Despite growing interest in children’s problematic media use, research examining the interplay among parental attitudes, specific and multi-dimensional parental mediation behaviors, and children’s problematic media use within dyadic parental contexts remains scarce, particularly in non-Western settings such as China. Furthermore, the distinct role of fathers relative to mothers remains underexamined. Addressing these gaps, the present study aimed to investigate the associations among parental attitudes, specific mediation behaviors across eight domains, and young children’s problematic media use within Chinese families. An Actor–Partner Interdependence Mediation Model (APIMeM) framework was employed to analyze these complex pathways (see Figure 1).

Specifically, building upon the reviewed literature and theoretical frameworks, we proposed the following hypotheses:

First, based on the established protective function of parental involvement, we hypothesized that active mediation, restrictive mediation, co-use, and parental knowledge would negatively predict children’s problematic media use, but autonomy support would positively predict children’s problematic media use (Hypothesis 1) (Chang et al., 2019; E. J. Lee & Kim, 2018; Mitchell et al., 2003; Su et al., 2018; Vandewater et al., 2005).

Second, we hypothesized that parents’ more positive attitudes toward screen use would negatively predict their own restrictive mediation but positively predict their active mediation, co-use, autonomy support, and parental knowledge (Hypothesis 2a) (Cohen et al., 2023; H. E. Lee et al., 2022; Nikken & Schols, 2015). We also hypothesized that parents’ more positive attitudes toward screen use would negatively predict their partner’s restrictive mediation but positively predict their partner’s active mediation, co-use, autonomy support, and parental knowledge (Hypothesis 2b) (APIM; Kenny et al., 2020).

Finally, integrating these pathways, we hypothesized that parents’ more positive attitudes toward screen use would positively influence children’s PMU via their impact on restrictive mediation but negatively influence children’s PMU via their impact on active mediation, co-use, autonomy support, and parental knowledge, considering both actor and partner mediation pathways within the model (Hypothesis 3) (IT-CPU; Domoff et al., 2020).

## 2. Materials and Methods

### 2.1. Participants

This study analyzed data drawn from a larger research project investigating how the family digital environment influences early childhood development in urban areas of South China. Ethical approval was granted by the institutional review board of the corresponding author’s affiliated institution, and informed consent was obtained from all participating families.

Initially, an online survey (via Wenjuanxing, China) was distributed by cooperating kindergartens to parents. After providing informed consent, parents independently completed the surveys. To accurately match parent dyads within the same household, each participant provided their own and their partner’s phone numbers as unique identifiers. After excluding 130 cases due to children’s ages falling outside the predefined range (3–6 years), the final analytic sample comprised 1802 matched parent dyads.

The children’s average age was 5.15 years (SD = 0.81), and 54.7% were boys. More than half of the parents held a bachelor’s degree or higher (mothers: 57.8%; fathers: 60.4%), and the majority of families (74.9%) reported an annual household income exceeding 100,000 RMB. Detailed demographic information is summarized in Table 1. Notably, the ‘Unemployed’ category captured a broader group than those actively seeking work, an important distinction for the parents in our sample.

### 2.2. Measures

#### 2.2.1. Parents’ Attitude Toward Screen Use

The parents’ attitudes toward screen use (PASUs) scale, originally developed by Pila et al. (2021), was adapted to measure parents’ attitudes regarding their children’s media usage. To better align with the early childhood context, the term “technology” was replaced with “media use.” The scale was translated into Mandarin using a forward–back-translation procedure by bilingual researchers to ensure linguistic and conceptual equivalence. The adapted scale consisted of five items (e.g., “Media use can help develop children’s critical thinking skills” and “Media use is useful for social interactions among children”), each rated on a 4-point Likert-type scale (1 = strongly disagree; 4 = strongly agree). Higher scores indicate more positive parental attitudes toward children’s screen use. In the current study, Cronbach’s alpha coefficients demonstrated good internal consistency (fathers: α = 0.85; mothers: α = 0.84). Confirmatory factor analysis showed excellent fits for fathers (χ^2^(5) = 66.93, CFI = 0.98, TLI = 0.97, RMSEA = 0.08, 90% CI [0.07, 0.10], SRMR = 0.02) and mothers (χ^2^(5) = 113.93, CFI = 0.97, TLI = 0.93, RMSEA = 0.11, 90% CI [0.09, 0.13], SRMR = 0.03).

#### 2.2.2. Parental Mediation Behaviors

Both fathers and mothers completed the 20-item parental mediation behavior (PMB) scale, adapted from (Zhou et al., 2022). The original scale included one item per domain; in this study, each domain was expanded to 2–3 items to enhance content coverage and reliability. Minor wording adjustments were made for clarity and consistency (e.g., “smartphone” → “digital device”). The PMB assesses eight dimensions: active mediation–communication frequency (e.g., “I ask my child what activities they do with digital devices”), active mediation–communication quality (e.g., “When I discuss topics related to digital device use with my child, they listen to me”), restrictive mediation–hands-on monitoring (e.g., “I limit the amount of time my child spends using digital devices”), restrictive mediation–sequence control (e.g., “I have clear reward measures for my child’s digital device behaviors”), restrictive mediation–acquaintance monitoring (e.g., “I ask grandparents and other relatives to help monitor my child’s use of digital devices”), autonomy support (e.g., “I allow my child to independently decide which digital devices to use and how to use them”), co-use (e.g., “I watch videos or listen to music on digital devices together with my child”), and parental knowledge (e.g., “I am aware of how long my child uses digital devices”).

Confirmatory factor analysis showed excellent fits for fathers (χ^2^(142) = 698.62, χ^2^/df = 4.92, CFI = 0.97, TLI = 0.96, RMSEA = 0.05, 90% CI [0.04, 0.05], SRMR = 0.04) and mothers (χ^2^(142) = 757.57, χ^2^/df = 5.34, CFI = 0.97, TLI = 0.95, RMSEA = 0.05, 90% CI [0.05, 0.05], SRMR = 0.04). Cronbach’s alpha indicated good internal consistency for fathers (α = 0.78–0.89) and mothers (α = 0.79–0.91), except moderate reliability for the co-use dimension (fathers: α = 0.52; mothers: α = 0.50).

#### 2.2.3. Problematic Media Use

Children’s problematic media use was assessed using the Problematic Media Use Scale—Short Form (PMU-SF; Domoff et al., 2019), a 9-item parent-report measure completed by mothers. The scale covers multiple dimensions of problematic use, including impaired control, withdrawal, and psychosocial consequences (e.g., “It is hard for my child to stop using screen media,” “My child becomes frustrated when he/she cannot use screen media,” and “My child’s screen media use interferes with family activities”). Items were scored on a 5-point Likert-type scale ranging from 1 (strongly disagree) to 5 (strongly agree), with higher scores indicating more severe problematic media use in children. In the current study, Cronbach’s alpha indicated excellent internal consistency (α = 0.90).

#### 2.2.4. Covariates

Covariates included child age (in years), child gender (1 = male, 2 = female), number of children in the family (1 to 5; responses > 5 recoded as invalid), and family socioeconomic status (SES). The family SES score was computed as the arithmetic mean of standardized scores for highest parental education, highest parental occupational status, and annual family income, following OECD’s PISA conventions (OECD, 2024). Higher scores represent higher family SES.

### 2.3. Analysis Strategy

Descriptive analyses were conducted using R (version 4.4.1; RStudio IDE). Given minimal missing data (0.3%, only on “number of children”), full information maximum likelihood (FIML) was applied.

Prior to testing the main hypotheses, a series of preliminary analyses were conducted in Mplus 8.11 (Muthén & Muthén, 2018) to establish the statistical assumptions and a valid baseline model. This process involved confirming measurement invariance (configural, metric and scalar) to ensure constructs were measured equivalently across parents, testing for dyad distinguishability, and employing equality constraints on paths to finalize the most parsimonious model structure (Cook & Kenny, 2005; Kenny & Ledermann, 2010; Kim & Kim, 2022; Sakaluk et al., 2021).

The primary analysis then tested the hypothesized pathways using an Actor–Partner Interdependence Mediation Model (APIMeM) by incorporating children’s problematic media use (PMU) as the outcome variable, retaining the established constraints and all covariates. Due to potentially elevated Type I error rates associated with bootstrapping in complex structural equation models, indirect effects and their 95% confidence intervals (CIs) were estimated using Monte Carlo sampling with 10,000 replications via the Rmediation package (Tofighi & MacKinnon, 2011). Mediation was determined to be significant if the corresponding CIs excluded zero.

Model fit was evaluated using recommended criteria: CFI and TLI > 0.90, and RMSEA and SRMR < 0.08 (Kline, 2016). Chi-square difference tests (Δχ^2^) were primarily used for model comparisons in APIM analyses due to their sensitivity in detecting local misfits with small degree-of-freedom changes (Barrett, 2007; Hayduk et al., 2007). Changes in CFI (<0.01) and RMSEA (<0.015) provided supplementary context for assessing model fit alterations (F. F. Chen, 2007).

## 3. Results

### 3.1. Primary Analyses

Correlation analyses and descriptive statistics for all key variables are summarized in Table 2. The main findings were as follows: (1) Mothers’ PASUs were significantly and positively correlated with their own PMB, particularly active mediation strategies (frequency and quality), autonomy support, co-use, and parental knowledge; restrictive mediation strategies showed no significant correlations. (2) Mothers’ PASUs showed limited correlations with fathers’ PMB, with significant associations observed primarily for co-use and autonomy support. (3) Fathers’ PASUs were significantly and positively related to their own PMB, particularly active communication quality, autonomy support, co-use, and several restrictive mediation practices. However, these associations were generally weaker than those observed for mothers. (4) Fathers’ PASUs showed minimal significant associations with mothers’ PMB, primarily restricted to co-use. (5) Both mothers’ and fathers’ PASUs were significantly correlated with children’s problematic media use; notably, fathers’ PASUs demonstrated weaker correlations than mothers’ PASUs. (6) PMBs from both parents, particularly active mediation quality, restrictive mediation strategies (hands-on monitoring and sequence control), and parental knowledge, were significantly negatively associated with children’s problematic media use.

### 3.2. Mediation Role of PMB in PASU and PMU

Prior to the main analysis, measurement invariance for the PASUs and PMB scales was established across mothers and fathers. Metric invariance was met for both scales, indicating that parents interpreted the constructs similarly. Furthermore, the latent means for PASU and PMB did not significantly differ between parents, implying that the overall levels of these attitudes and behaviors were equivalent across the dyad (details in Table A1).

Subsequently, the baseline Actor–Partner Interdependence Model (APIM) was established. A distinguishability test confirmed that mothers and fathers function as statistically distinguishable dyad members (Δχ^2^ (17) = 29.40, *p* < 0.05). For model parsimony, equality constraints were tested across paths, showing that all eight actor-effect paths and five of the eight partner-effect paths were equivalent across parents. However, three partner paths—assessing the influence of a parent’s attitude on their partner’s communication quality, hands-on monitoring, and parental knowledge—differed significantly between parents and were thus freely estimated. This partially constrained model was retained as the baseline for the main mediation analysis (details in Table A2).

**Figure 2:**
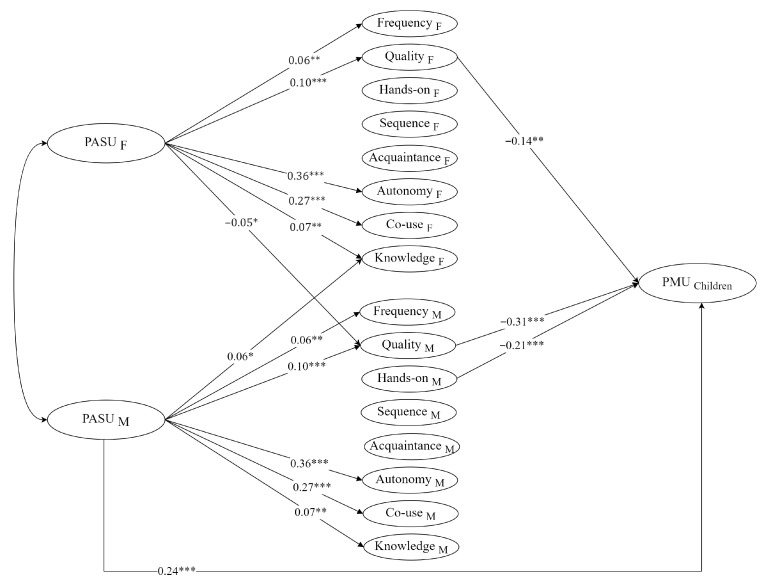
Unstandardized APIMeM model linking parents’ attitude toward screen use to children’s problematic media use via parental mediation behaviors. Note: F = Father, M = Mother. Number of children in the family, child age, child gender, and family SES are included as covariates. For clarity, non-significant regression paths, latent variable indicators, and the correlation between domains of PMB are not displayed. * *p* < 0.05, ** *p* < 0.01, *** *p* < 0.001.

To assess whether PASUs affect children’s PMU via specific mediation behaviors, we extended the baseline APIM to include PMU and sequentially tested equality constraints on each mother- and father-path (details in Table A3). Model comparisons showed that equating communication frequency, sequence control, acquaintance monitoring, autonomy support, co-use, and parental knowledge did not impair the fit (all Δχ^2^ n.s., details in Table A4). Thus, a model with equality constraints in these paths was selected as the best model. The final mediation model is shown in Figure 2.

As shown in Figure 2 (see detailed coefficients in Table A5), higher communication quality predicted lower levels of children’s PMU for both fathers (β = –0.14, *p* = 0.001) and mothers (β = –0.31, *p* < 0.001). In addition, mothers’ hands-on monitoring was inversely associated with PMU (β = –0.21, *p* < 0.001), whereas the corresponding paternal path was non-significant.

After these behaviors were taken into account, a notable direct effect remained: mothers’ favorable attitudes were still positively related to PMU, suggesting an unmediated pathway through which maternal permissiveness may foster heavier or less regulated device use.

For the mediation effects, as shown in Table 3, Monte-Carlo confidence intervals confirmed communication quality as the only reliable mediator. Fathers’ positive attitudes lowered children’s PMU through their own higher-quality conversations (indirect = −0.01, 95% CI [−0.03, −0.01]) but simultaneously raised PMU by diminishing the quality of mothers’ conversations (indirect = 0.02, 95% CI [0.00, 0.03]). As for mothers, their favorable attitudes reduced PMU via their own enhanced communication quality (indirect = −0.03, 95% CI [−0.05, −0.02]).

## 4. Discussion

This study used an APIM-mediation framework to clarify how Chinese mothers’ and fathers’ PASUs translate into children’s PMU through specific parenting behaviors. Consistent actor effects showed that more favorable attitudes were associated with higher levels of their own media parenting behaviors, including active mediation (both communication frequency and quality), autonomy support, co-use, and parental knowledge, but not restrictive mediation behaviors. Partner influence was modest and asymmetric: mothers’ positive attitudes increased fathers’ knowledge of the child’s screen activities, while fathers’ positive attitudes diminished mothers’ communication quality. Of all PMB dimensions, only higher communication quality (both parents) and mothers’ hands-on monitoring directly related to lower PMU. Mediation effect tests confirmed communication quality as the sole reliable mediator: each parent’s own favorable attitudes reduced PMU by boosting their communication quality, and fathers’ attitudes additionally increased PMU by eroding mothers’ communication quality. Mothers’ attitudes also retained a direct positive link with PMU, signaling an unmediated risk pathway beyond the parenting behaviors measured here.

The following is a brief note on the study’s analytical approach: While most paths in our model were theoretically guided, the latent mean comparisons between parents were exploratory. These analyses revealed no significant differences in parental media attitudes or mediation behaviors. A likely explanation lies in our sample’s demographic profile—predominantly highly educated, higher-income urban parents, over half of whom held at least a bachelor’s degree. Prior research indicates that such parents typically demonstrate higher digital competence and more balanced, opportunity-oriented media attitudes, which in turn are linked to more consistent mediation practices across caregivers (Lou et al., 2024; Rasmussen et al., 2016).

### 4.1. Actor and Partner Effects in Parental Attitudes and Behaviors

Consistent with prior domain-specific research, each parent’s positive attitude toward young children’s screen use significantly predicted greater engagement in active mediation (communication frequency and quality), autonomy support, co-use, and higher parental knowledge. These findings mirror evidence where parents who see digital media as beneficial tend to talk with children about content and explore it together rather than simply control it (Lauricella et al., 2015; Nikken & Schols, 2015).

By contrast, parental attitudes were not linked to the restrictive strategies of sequence control, hands-on monitoring, or acquaintance monitoring. First, from a psychological–cognitive perspective, parenting behaviors often stem from two distinct mindsets: restrictive actions are typically driven by a “risk-avoidance” framework aimed at preventing potential harm (Nathanson, 2001; Nikken & Jansz, 2006), whereas the positive attitudes measured in this study reflect an “opportunity-seeking” framework (Nikken & Jansz, 2006). In line with the Theory of Planned Behavior, these divergent cognitive pathways naturally result in a lack of a direct association between a positive, benefit-oriented attitude and a specific, risk-mitigating behavior (Rasmussen et al., 2016). Second, Chinese preschoolers already experience relatively tight screen-time limits as part of everyday guan parenting (Chao, 1994). This cultural script promotes a high baseline level of restrictive mediation across the sample, reducing behavioral variability and thus statistically attenuating the predictive power of individual attitudes. Finally, at the family systems level, acquaintance monitoring is driven more by logistical arrangements, particularly the widespread reliance on grandparents for daytime childcare in urban China, making this practice a necessity dictated by family structure rather than a discretionary choice driven by parents’ personal media attitudes (F. Chen et al., 2011). Together, the non-significant paths underline that parents’ positive views of media translate chiefly into engagement-oriented behaviors rather than into control-oriented ones.

Our dyadic analysis also revealed asymmetric—but complementary—partner effects. First, mothers’ favorable screen-media attitudes increased fathers’ knowledge of the child’s digital activities. This spill-over is consistent with evidence that mothers often coordinate information flow about daily routines, thereby equipping fathers with the “facts” they need to monitor effectively. In contrast, fathers’ attitudes did not significantly enhance mothers’ knowledge, which may reflect mothers’ already high baseline awareness of their children’s media use, reducing the incremental influence of paternal attitudes. (Daminger, 2019; Stevens, 1988). Second—and in the opposite direction—fathers’ favorable attitudes were associated with lower communication quality reported by mothers. One plausible scenario is a de facto “division of digital labor”. Research indicated that males tend to exhibit greater proficiency in technical operations (e.g., software installation, network configuration) compared to females (Hargittai & Shafer, 2006). When fathers demonstrate higher technical competence, mothers often reallocate technology-related parenting responsibilities to them, leading mothers to step back from detailed conversations with the child and to channel media-related information toward the father instead (“he’s got this”). Such cross-parent adjustments echo family systems’ notions of role complementarity and crossover (Minuchin, 1985) and align with research showing that fathers’ increased domain-specific engagement can prompt maternal withdrawal from that same domain (Fagan & Barnett, 2003; Feinberg, 2003).

These asymmetries can also be understood through differences in parenting stress and the nature of information transfer. Mothers, who typically report higher parenting stress (Abidin et al., 2006; Rusu et al., 2025; Trumello et al., 2023), may perceive fathers’ active involvement in managing children’s screen time as a relief signal, prompting them to reduce their own direct engagement (Nomaguchi et al., 2017). In contrast, fathers’ behavior appears less influenced by mothers’ attitudes because involvement—especially in communication—is largely self-determined, rooted in one’s own confidence and motivation, rather than easily shaped by a partner’s cues. This distinction between “knowledge,” which is readily transmissible across parents, and “involvement,” which depends on self-determined commitment, helps explain why maternal attitudes increase paternal knowledge but paternal attitudes do not similarly enhance maternal engagement.

### 4.2. The Role of Media-Related Parenting Behaviors in Children’s Problematic Media Use

The current study revealed that specific media-related parenting behaviors significantly predict children’s problematic media use. Notably, both fathers’ and mothers’ active communication quality was found to negatively predict children’s PMU. This aligns with a body of literature suggesting that open, frequent, and supportive discussions about media content, rules, and online safety are crucial components of effective parental mediation, fostering critical thinking and understanding in children rather than relying solely on rule imposition (Dedkova & Mýlek, 2023; Liu et al., 2023). Furthermore, mothers’ restrictive hands-on mediation also emerged as a significant negative predictor of children’s PMU. This finding is consistent with research indicating that for preschoolers, who possess limited self-regulation skills, certain forms of restrictive mediation, such as direct monitoring and rule-setting, can be effective in mitigating problematic use (Shawcroft et al., 2023). The effectiveness of such hands-on approaches employed by mothers within the Chinese cultural context can also be interpreted through the lens of guan parenting, which emphasizes attentive oversight and careful governance (Chan et al., 2009).

Conversely, several other PMB dimensions measured in this study—such as fathers’ hands-on monitoring, sequence control, acquaintance monitoring, autonomy support, co-use, and parental knowledge—did not emerge as significant direct predictors of children’s PMU. These could also be seen in the literature, where restrictive mediation sometimes shows limited or even counterproductive effects, particularly if perceived as overly controlling or inconsistently applied (Vossen et al., 2024). Similarly, co-use, if predominantly passive or not involving active discussion, has been linked to increased PMU in some studies (Nathanson, 2001; Paavonen et al., 2009). Furthermore, the finding that autonomy support was not significantly related to PMU in preschoolers is developmentally plausible; young children’s cognitive immaturity and limited self-regulatory capacities mean that high levels of autonomy in media choices without adequate structure may not be protective and could even be detrimental (Fitzpatrick et al., 2022). Parental knowledge about media, while important, may not translate into reduced PMU if not accompanied by specific, effectively implemented behaviors (Shawcroft et al., 2024). Comparisons to broader meta-analyses (e.g., Collier et al., 2016; Nikken & Jansz, 2014) should consider the current study’s nuanced PMB measures, the unique developmental needs of preschoolers, and the influence of culturally specific parenting practices such as guan within the Chinese context (Chao, 1994).

A key finding of this study is the significant mediating role of parental communication quality in the relationship between PASU and children’s PMU. This suggests that parental attitudes do not directly influence child outcomes but operate indirectly through the specific parenting behavior of communication quality (Nathanson, 2001; Valkenburg et al., 2013). Regarding actor effects, both mothers’ and fathers’ PASUs indirectly predicted children’s PMU via their respective communication quality. This underscores that a parent’s beliefs about media shape their interactions and conversations with their child, subsequently influencing the child’s media use patterns (Ajzen, 1991; Nikken & Schols, 2015). However, an additional partner pathway ran in the opposite direction: fathers’ favorable attitudes dampened mothers’ communication quality, which then predicted higher PMU, producing a small but significant risk effect. This pattern aligns with the work of family systems showing that one parent’s expanded role in a domain can prompt compensatory withdrawal by the other (Feinberg, 2003). No other mediation behavior carried the influence of parental attitudes to PMU, underscoring the centrality of how parents talk with their child—rather than how often they talk, set limits, or co-use devices—in explaining early problematic media use.

### 4.3. Maternal Positive Attitudes and Child Problematic Media Use

A particularly noteworthy finding from this study is the direct positive association between mothers’ favorable attitudes toward media use and higher levels of their children’s problematic media use, an effect that persisted even after accounting for the mediating influence of the measured media-related parenting behaviors. This suggests that a mother’s positive orientation toward media, in and of itself, may contribute to increased PMU in preschoolers through mechanisms not fully captured by the assessed parenting strategies. Mothers with a more permissive attitude may allow more frequent or longer screen exposure or use devices as a calming tool—conditions known to foster compulsive engagement in early childhood (Çaylan et al., 2021; H. E. Lee et al., 2022). Prior research similarly indicates that parents holding affirmative views about mobile devices typically impose fewer limits, leading to substantially higher screen time for their children (Cingel & Krcmar, 2013). Additionally, mothers with highly favorable views toward media might themselves model elevated media consumption or a more permissive engagement style, behaviors that preschool-aged children are especially prone to emulate (Nikken, 2017).

Critically, the results highlight that positive parental attitudes alone are insufficient in mitigating problematic use; rather, such attitudes must be complemented by effective mediation strategies to manage associated risks (Symons et al., 2017). This implies that positive attitudes toward media require balancing proactive oversight with clear boundaries.

### 4.4. Limitations and Future Research Directions

While this study provides valuable insights into the dyadic interplay of parental attitudes, mediation behaviors, and young children’s problematic media use within urban Chinese families, several limitations warrant acknowledgment, guiding future research.

A primary limitation is the cross-sectional nature of the data. Although the current model is grounded in the Theory of Planned Behavior (Ajzen, 1991) and the Interactional Theory of Childhood Problematic Media Use (Domoff et al., 2020), this design cannot establish causality or rule out reciprocal relationships. For instance, parental attitudes or mediation strategies might evolve in response to observed child media use or management challenges.

Secondly, the current study must be interpreted within the specific socio-cultural and demographic context of the sample: 1802 urban Chinese parent dyads with relatively high parental education and income. As Bornstein (2013, pp. 3–18) highlights, cultural frameworks profoundly shape parenting. Families with lower SES may also face distinct media challenges or hold different attitudes, while rural areas might exhibit unique family structures and values impacting media-related decisions. Therefore, future research should aim for broader representation across geographic, socioeconomic, and cultural contexts, including rural China, other East Asian populations, and cross-cultural comparisons.

Furthermore, the current study relies on maternal reports for the child’s PMU, creating an asymmetry in our dyadic design. This presents a key limitation. The direct correlation between mothers’ PASU and their reported child PMU suggests a potential for common method bias, where mothers with more positive media attitudes may be less inclined to perceive certain screen-related behaviors as problematic, or might underestimate their severity, influencing their PMU reports. This subjective perceptual difference could lead to measurement outcomes not fully reflecting objective behavior. To better understand the PASU-PMU relationship and mitigate reporting bias, future research should incorporate PMU assessments from both parents and consider adding more nuanced scale items and qualitative approaches, such as in-depth interviews, to enhance validity and capture more nuanced family media dynamics.

## 5. Conclusions

This study employed the Actor–Partner Interdependence Model (APIM) to provide a nuanced understanding of the complex relationships between parents’ attitudes toward screen use, their distinct parental mediation behaviors, and young children’s problematic media use within the context of urban Chinese families. Our findings underscore the pivotal role of parental factors, revealing significant actor effects, asymmetric partner effects, and crucial mediation pathways. Specifically, we found that parents’ positive attitudes were primarily associated with their own engagement in active and supportive mediation strategies (like communication, autonomy support, co-use, and knowledge acquisition) rather than restrictive ones. Among the eight mediation domains, communication quality and maternal hands-on monitoring uniquely predicted lower levels of children’s problematic media use, underscoring the dual importance of dialogic guidance and direct supervision in early childhood. Critically, active mediation—specifically communication quality—emerged as a significant mediator, linking both mothers’ and fathers’ positive attitudes to reduced child problematic media use (Clark, 2011; Valkenburg et al., 1999). Dyadic analyses further revealed an asymmetric influence: mothers’ positive attitudes enhanced fathers’ knowledge of the child’s screen activities; by contrast, fathers’ positive attitudes eroded mothers’ communication quality, thereby indirectly heightening children’s problematic media use, suggesting distinct parental roles and interdependence within the family system (Cox & Paley, 2003).

Together, these findings highlight the need for interventions that (a) cultivate high-quality parent–child conversation, (b) safeguard maternal dialogue from unintended paternal attenuation, and (c) foster complementary, rather than conflicting, dyadic strategies to curb early problematic media use.

## Figures and Tables

**Table 1:** Demographic information of the sample (N = 1802).

	M (SD) %	
Children’s age (years)	5.15 (0.809)	
Children’s gender		
Boy	985 (54.7%)	
Girl	817 (45.3%)	
Number of children	1.70 (0.566)	
Family annual income (RMB)		
Below 30,000	97 (5.4%)	
30,000–50,000	86 (4.8%)	
50,001–100,000	269 (14.9%)	
100,001–200,000	488 (27.1%)	
200,001–300,000	317 (17.6%)	
Above 300,001	545 (30.2%)	
Occupation	Mother	Father
Unemployed ^a^	368 (20.4%)	88 (4.9%)
Non-technical or semi-technical worker	47 (2.6%)	36 (2.0%)
Technical worker or semi-professional	236 (13.1%)	289 (16.0%)
Professional or officer	880 (48.8%)	829 (46.0%)
Medium level professional or administrator	224 (12.4%)	426 (23.6%)
High-level professional or administrator	47 (2.6%)	134 (7.4%)
Educational background		
Junior high school or below	100 (5.5%)	89 (4.9%)
Senior high school	216 (12.0%)	231 (12.8%)
Associate college degree	445 (24.7%)	394 (21.9%)
Bachelor’s degree	910 (50.5%)	914 (50.7%)
Master’s degree or above	131 (7.3%)	174 (9.7%)

Note: ^a^ This category includes parents who are out of work and seeking employment as well as those who identify as full-time homemakers or stay-at-home parents (Avvisati & Wuyts, 2024, p. 11).

**Table 2:** Means, standard deviations, and bivariate correlations of study variables (N = 1802).

Variable	1	2	3	4	5	6	7	8	9	10	11	12	13	14	15	16	17	18	19
1. PASU_Father	-																		
2. PASU_Mother	0.28 ***	-																	
3. Frequency_Father	0.07 **	0.03	-																
4. Quality_Father	0.13 ***	0.08 ***	0.54 ***	-															
5. HandsOn_Father	−0.02	0.05 *	0.40 ***	0.31 ***	-														
6. Sequence_Father	−0.01	−0.02	0.26 ***	0.30 ***	0.22 ***	-													
7. Acquaintance_Father	0.03	0.01	0.23 ***	0.26 ***	0.20 ***	0.47 ***	-												
8. Autonomy_Father	0.17 ***	0.03	0.08 **	0.11 ***	−0.08 ***	0.13 ***	0.17 ***	-											
9. CoUse_Father	0.24 ***	0.12 ***	0.23 ***	0.24 ***	0.13 ***	0.16 ***	0.20 ***	0.42 ***	-										
10. Knowledge_Father	0.10 ***	0.09 ***	0.40 ***	0.36 ***	0.42 ***	0.18 ***	0.17 ***	0.05 *	0.32 ***	-									
11. Frequency_Mother	−0.02	0.03	0.17 ***	0.12 ***	0.15 ***	0.06 **	0.08 ***	−0.05 *	0.06 **	0.12 ***	-								
12. Quality_Mother	−0.02	0.07 **	0.14 ***	0.18 ***	0.12 ***	0.07 **	0.05 *	−0.03	0.05 *	0.14 ***	0.57 ***	-							
13. HandsOn_Mother	−0.03	−0.02	0.14 ***	0.08 ***	0.21 ***	0.07 **	0.06 **	−0.04	0.02	0.15 ***	0.48 ***	0.36 ***	-						
14. Sequence_Mother	−0.05 *	−0.01	0.06 *	0.09 ***	0.09 ***	0.22 ***	0.09 ***	0.01	0.03	0.06 **	0.22 ***	0.30 ***	0.23 ***	-					
15. Acquaintance_Mother	−0.02	0.00	0.06 **	0.07 **	0.11 ***	0.12 ***	0.26 ***	0.02	0.04	0.05 *	0.17 ***	0.22 ***	0.22 ***	0.41 ***	-				
16. Autonomy_Mother	0.06 *	0.18 ***	−0.01	0.03	−0.01	0.02	0.01	0.17 ***	0.14 ***	−0.01	0.02	0.09 ***	−0.07 **	0.15 ***	0.16 ***	-			
17. CoUse_Mother	0.09 ***	0.20 ***	0.04	0.07 **	0.02	0.02	0.03	0.11 ***	0.23 ***	0.07 **	0.17 ***	0.21 ***	0.08 ***	0.15 ***	0.16 ***	0.41 ***	-		
18. Knowledge_Mother	−0.01	0.04	0.13 ***	0.08 **	0.12 ***	0.06 *	0.04	−0.01	0.08 **	0.14 ***	0.46 ***	0.34 ***	0.46 ***	0.22 ***	0.20 ***	0.04	0.28 ***	-	
19. PMU_Child	0.07 **	0.16 ***	−0.08 ***	−0.12 ***	−0.09 ***	−0.03	−0.01	0.08 **	0.03	−0.09 ***	−0.19 ***	−0.28 ***	−0.23 ***	−0.10 ***	−0.09 ***	0.09 ***	0.00	−0.19 ***	-
M	2.80	2.69	3.92	3.65	4.31	3.30	3.19	2.69	3.37	4.08	4.11	3.80	4.41	3.45	3.29	2.69	3.40	4.27	1.93
SD	0.66	0.66	0.67	0.69	0.61	1.01	1.08	1.06	0.88	0.65	0.68	0.70	0.59	1.00	1.12	1.12	0.91	0.65	0.67

Note: PASU = parents’ attitude toward screen use; PMU = children’s problematic media use. Frequency = active mediation − communication frequency; Quality = active mediation − communication quality; HandsOn = restrictive mediation − hands-on monitoring; Sequence = restrictive mediation − sequence control; Acquaintance = restrictive mediation − acquaintance monitoring; Autonomy = autonomy support; CoUse = co-use of media; Knowledge = parental knowledge. Suffixes _Father and _Mother indicate the responding parent. * *p* < 0.05, ** *p* < 0.01, *** *p* < 0.001.

**Table 3:** Unstandardized indirect effects of PASU on PMU via PMB.

Specific Indirect Pathways		From Father’s PASU			From Mother’s PASU		
		Estimate	SE	95% CI	Estimate	SE	95% CI
Via Father’s	Frequency	0.00	0.00	[−0.00, 0.01]	−0.00	0.00	[−0.01, 0.00]
	Quality	**−0.01**	**0.01**	**[−0.03, −0.01]**	−0.01	0.00	[−0.01, 0.00]
	Hands-on	0.00	0.00	[−0.00, 0.00]	0.00	0.00	[−0.01, 0.00]
	Sequence	0.00	0.00	[−0.00, 0.00]	0.00	0.00	[−0.00, 0.00]
	Acquaintance	0.00	0.00	[−0.00, 0.00]	0.00	0.00	[−0.00, 0.00]
	Autonomy	0.02	0.01	[−0.00, 0.04]	−0.00	0.00	[−0.01, 0.00]
	Co-use	0.00	0.02	[−0.03, 0.04]	0.00	0.00	[−0.00, 0.01]
	Knowledge	−0.00	0.00	[−0.01, 0.00]	−0.00	0.00	[−0.01, 0.00]
Via Mother’s	Frequency	−0.00	0.00	[−0.01, 0.00]	0.00	0.00	[−0.00, 0.01]
	Quality	**0.02**	**0.01**	**[0.00, 0.03]**	**−0.03**	**0.01**	**[−0.05, −0.02]**
	Hands-on	0.00	0.01	[−0.01, 0.01]	0.01	0.00	[−0.00, 0.01]
	Sequence	0.00	0.00	[−0.00, 0.00]	0.00	0.00	[−0.00, 0.00]
	Acquaintance	0.00	0.00	[−0.00, 0.00]	0.00	0.00	[−0.00, 0.00]
	Autonomy	−0.00	0.00	[−0.01, 0.00]	0.02	0.01	[−0.00, 0.04]
	Co-use	0.00	0.00	[−0.00, 0.01]	0.00	0.02	[−0.03, 0.04]
	Knowledge	0.00	0.00	[−0.00, 0.01]	−0.00	0.00	[−0.01, 0.00]

Note: The bold indirect statistics are significant based on the Monte Carlo 95% CI.

## Data Availability

The raw data supporting the conclusions of this article will be made available by the authors on request.

## Appendix A

**Table A1:** Goodness-of-fit statistics for measurement invariance tests of PASU and PMB.

Variable	Models	χ^2^ (*df*)	CFI	TLI	RMSEA	SRMR	Δχ^2^ (Δ*df*)	ΔCFI	ΔRMSEA
PASU	Configural	180.85 (10)	0.98	0.95	0.10	0.02	–	–	–
	Metric	184.14 (14)	0.98	0.96	0.08	0.03	3.29 (4)	0.00	−0.02
	Scalar	194.90 (18)	0.97	0.97	0.07	0.03	10.76 (4) *	0.01	−0.01
	Mean	220.95 (19)	0.97	0.97	0.08	0.04	26.05 (1) ***	0.00	0.01
PMB	Configural	1456.19 (284)	0.97	0.96	0.05	0.04	–	–	–
	Metric	1472.63 (296)	0.97	0.96	0.05	0.04	16.44 (12)	0.00	0.00
	Scalar	1527.10 (308)	0.97	0.96	0.05	0.04	54.46 (12) ***	0.00	0.00
	Mean	1633.66 (315)	0.96	0.96	0.05	0.05	106.56 (7) ***	0.01	0.00

Note: χ^2^ = chi-square; *df* = degrees of freedom; CFI = comparative fit index; TLI = Tucker–Lewis index; RMSEA = root-mean-square error of approximation. * *p* < 0.05, *** *p* < 0.001.

**Table A2:** Goodness-of-fit statistics for sequential equality constraint in APIM (N = 1802).

Models	Constraints	χ^2^ (*df*)	CFI	TLI	RMSEA	SRMR	Δχ^2^ (Δ*df*)	ΔCFI	ΔRMSEA
Baseline	-	3030.00 (1150)	0.96	0.95	0.03	0.03	-	-	-
Distinguishability	φ_1_ = φ_2_ ψ_11–18_ = ψ_21–28_ a_11–18_ = a_21–28_ p_11–18_ = p_21–28_	3059.40 (1167)	0.96	0.95	0.03	0.03	29.40 (17) *	0.00	0.00
Fixed Actor-Effect	a_11_ = a_21_	3030.69 (1151)	0.96	0.95	0.03	0.03	0.69 (1)	0.00	0.00
	a_12_ = a_22_	3030.23 (1151)	0.96	0.95	0.03	0.03	0.24 (1)	0.00	0.00
	a_13_ = a_23_	3030.45 (1151)	0.96	0.95	0.03	0.03	0.45 (1)	0.00	0.00
	a_14_ = a_24_	3030.04 (1151)	0.96	0.95	0.03	0.03	0.04 (1)	0.00	0.00
	a_15_ = a_25_	3030.80 (1151)	0.96	0.95	0.03	0.03	0.81 (1)	0.00	0.00
	a_16_ = a_26_	3030.07 (1151)	0.96	0.95	0.03	0.03	0.08 (1)	0.00	0.00
	a_17_ = a_27_	3031.51 (1151)	0.96	0.95	0.03	0.03	1.51 (1)	0.00	0.00
	a_18_ = a_28_	3030.40 (1151)	0.96	0.95	0.03	0.03	0.41 (1)	0.00	0.00
Fixed Partner-Effect	p_11_ = p_21_	3031.60 (1151)	0.96	0.95	0.03	0.03	1.61 (1)	0.00	0.00
	p_12_ = p_22_	3039.16 (1151)	0.96	0.95	0.03	0.03	9.17 (1) **	0.00	0.00
	p_13_ = p_23_	3037.84 (1151)	0.96	0.95	0.03	0.03	7.85 (1) **	0.00	0.00
	p_14_ = p_24_	3030.80 (1151)	0.96	0.95	0.03	0.03	0.80 (1)	0.00	0.00
	p_15_ = p_25_	3030.00 (1151)	0.96	0.95	0.03	0.03	0.00 (1)	0.00	0.00
	p_16_ = p_26_	3030.47 (1151)	0.96	0.95	0.03	0.03	0.48 (1)	0.00	0.00
	p_17_ = p_27_	3030.09 (1151)	0.96	0.95	0.03	0.03	0.09 (1)	0.00	0.00
	p_18_ = p_28_	3037.44 (1151)	0.96	0.95	0.03	0.03	7.44 (1) **	0.00	0.00
**Fixed Actor–Partner**	**Adopted**	**3041.58 (1163)**	**0.96**	**0.95**	**0.03**	**0.03**	**11.58 (13)**	**0.00**	**0.00**

Note: φ_1/2_ refers to the variance of PASUs for the father and mother, respectively. ψ_11–18/21–28_ refers to the residual variance of PMB for the father and mother, respectively. a_11–18_/a_21–28_ indicates actor effects (own PASU on own PMB); p_11–18_ indicates the father’s PASU on the mother’s PMBs, and p_21–28_ indicates the mother’s PASU on the father’s PMB. The adopted model incorporates all tenable equality constraints for actor and partner effects, as detailed in the main text. The model in bold is the best fit model for the following analysis. * *p* < 0.05, ** *p* < 0.01.

**Table A3:** Detailed unstandardized path coefficients for the APIM of parental attitudes toward screen use (PASUs) predicting parental mediation behaviors (PMBs).

Type	Predictor	Outcome	b	SE	*p*
Father to Father	PASU _Father_	Frequency _Father_	0.06 ***	0.02	0.003
		Quality _Father_	0.10 ***	0.02	<0.001
		Hands-on _Father_	−0.03	0.02	0.101
		Sequence _Father_	0.00	0.03	0.924
		Acquaintance _Father_	0.03	0.03	0.330
		Autonomy _Father_	0.36 ***	0.03	<0.001
		Co-use _Father_	0.27 ***	0.02	<0.001
		Knowledge _Father_	0.07 ***	0.02	0.001
Father to Mother	PASU _Father_	Frequency _Mother_	−0.03	0.02	0.210
		Quality _Mother_	−0.05 *	0.02	0.028
		Hands-on _Mother_	−0.02	0.02	0.345
		Sequence _Mother_	−0.05	0.03	0.083
		Acquaintance _Mother_	−0.02	0.03	0.432
		Autonomy _Mother_	−0.02	0.03	0.439
		Co-use _Mother_	0.03	0.02	0.190
		Knowledge _Mother_	−0.03	0.03	0.277
Mother to Father	PASU _Mother_	Frequency _Father_	−0.03	0.02	0.210
		Quality _Father_	0.04	0.02	0.087
		Hands-on _Father_	0.04	0.02	0.079
		Sequence _Father_	−0.05	0.03	0.083
		Acquaintance _Father_	−0.02	0.03	0.432
		Autonomy _Father_	−0.02	0.03	0.439
		Co-use _Father_	0.03	0.02	0.190
		Knowledge _Father_	0.06 *	0.03	0.022
Mother to Mother	PASU _Mother_	Frequency _Mother_	0.06 ***	0.02	0.003
		Quality _Mother_	0.10 ***	0.02	<0.001
		Hands-on _Mother_	−0.03	0.02	0.101
		Sequence _Mother_	0.00	0.03	0.924
		Acquaintance _Mother_	0.03	0.03	0.330
		Autonomy _Mother_	0.36 ***	0.03	<0.001
		Co-use _Mother_	0.27 ***	0.02	<0.001
		Knowledge _Mother_	0.07 ***	0.02	0.001
Covariates	Child age	Frequency _Father_	0.01	0.02	0.685
		Quality _Father_	0.04 *	0.02	0.022
		Hands-on _Father_	−0.02	0.02	0.291
		Sequence _Father_	−0.01	0.03	0.810
		Acquaintance _Father_	−0.03	0.03	0.256
		Autonomy _Father_	0.07 *	0.03	0.018
		Co-use _Father_	0.00	0.02	0.892
		Knowledge _Father_	−0.03	0.02	0.164
		Frequency _Mother_	0.01	0.02	0.540
		Quality _Mother_	0.02	0.02	0.165
		Hands-on _Mother_	−0.02	0.02	0.311
		Sequence _Mother_	0.01	0.03	0.621
		Acquaintance _Mother_	−0.05	0.03	0.085
		Autonomy _Mother_	0.06	0.03	0.070
		Co-use _Mother_	−0.04	0.02	0.107
		Knowledge _Mother_	−0.05 *	0.02	0.011
	Child gender	Frequency _Father_	−0.05	0.03	0.114
		Quality _Father_	−0.02	0.03	0.461
		Hands-on _Father_	−0.02	0.03	0.443
		Sequence _Father_	0.00	0.05	0.987
		Acquaintance _Father_	0.01	0.04	0.890
		Autonomy _Father_	−0.04	0.05	0.408
		Co-use _Father_	0.02	0.03	0.489
		Knowledge _Father_	−0.03	0.03	0.289
		Frequency _Mother_	0.01	0.03	0.784
		Quality _Mother_	0.00	0.03	0.901
		Hands-on _Mother_	0.00	0.03	0.952
		Sequence _Mother_	−0.10 *	0.05	0.029
		Acquaintance _Mother_	−0.04	0.05	0.345
		Autonomy _Mother_	−0.01	0.05	0.853
		Co-use _Mother_	−0.06	0.04	0.071
		Knowledge _Mother_	−0.08 *	0.03	0.010
	Num. of Children	Frequency _Father_	−0.10 ***	0.03	0.001
		Quality _Father_	−0.07 *	0.02	0.007
		Hands-on _Father_	−0.07 ***	0.02	0.003
		Sequence _Father_	0.03	0.04	0.517
		Acquaintance _Father_	−0.02	0.04	0.539
		Autonomy _Father_	0.03	0.04	0.517
		Co-use _Father_	−0.10 ***	0.03	0.003
		Knowledge _Father_	−0.10 ***	0.03	0.001
		Frequency _Mother_	−0.11 ***	0.03	<0.001
		Quality _Mother_	−0.03	0.03	0.184
		Hands-on _Mother_	−0.06 *	0.02	0.011
		Sequence _Mother_	0.12 ***	0.04	0.003
		Acquaintance _Mother_	0.08	0.04	0.068
		Autonomy _Mother_	0.01	0.05	0.805
		Co-use _Mother_	−0.05	0.03	0.099
		Knowledge _Mother_	−0.05	0.03	0.056
	SES	Frequency _Father_	0.01	0.02	0.422
		Quality _Father_	0.01	0.01	0.492
		Hands-on _Father_	0.01	0.01	0.442
		Sequence _Father_	−0.04	0.02	0.085
		Acquaintance _Father_	−0.10 ***	0.02	<0.001
		Autonomy _Father_	−0.06 *	0.02	0.014
		Co-use _Father_	−0.01	0.02	0.616
		Knowledge _Father_	0.02	0.01	0.124
		Frequency _Mother_	0.09 ***	0.02	<0.001
		Quality _Mother_	0.07 ***	0.01	<0.001
		Hands-on _Mother_	0.06 ***	0.01	<0.001
		Sequence _Mother_	0.00	0.02	0.822
		Acquaintance _Mother_	−0.06 **	0.02	0.005
		Autonomy _Mother_	−0.04	0.03	0.174
		Co-use _Mother_	0.01	0.02	0.496
		Knowledge _Mother_	0.08 ***	0.01	<0.001

Note: * *p* < 0.05, ** *p* < 0.01, *** *p* < 0.001.

**Table A4:** Goodness-of-fit statistics for equality constraint tests of paths from parental mediation behaviors (PMBs) to child problematic media use (PMU) in the mediation model.

Models	χ^2^ (*df*)	CFI	TLI	RMSEA	SRMR	Δχ^2^	ΔCFI	ΔRMSEA
Freely estimate	4668.17 (1654)	0.94	0.93	0.03	0.03	-	-	-
Frequency	4668.26 (1655)	0.94	0.93	0.03	0.03	0.09 (1)	0.00	0.00
Quality	4673.09 (1655)	0.94	0.93	0.03	0.03	4.92 (1) *	0.00	0.00
Hands-on	4672.11 (1655)	0.94	0.93	0.03	0.03	3.94 (1) *	0.00	0.00
Sequence	4668.23 (1655)	0.94	0.93	0.03	0.03	0.06 (1)	0.00	0.00
Acquaintance	4668.70 (1655)	0.94	0.93	0.03	0.03	0.53 (1)	0.00	0.00
Autonomy	4668.18 (1655)	0.94	0.93	0.03	0.03	0.01 (1)	0.00	0.00
Co-use	4668.18 (1655)	0.94	0.93	0.03	0.03	0.00 (1)	0.00	0.00
Knowledge	4668.60 (1655)	0.94	0.93	0.03	0.03	0.43 (1)	0.00	0.00
Direct effects	4685.72 (1655)	0.94	0.93	0.03	0.03	17.55 (1) ***	0.00	0.00
Adopted	4670.23 (1660)	0.94	0.93	0.03	0.03	2.06 (6)	0.00	0.00

Note: * *p* < 0.05, *** *p* < 0.001.

**Table A5:** Detailed unstandardized coefficients for the Actor–Partner Interdependence Mediation Model.

Path Type	Predictor	Outcome	b	SE	*p*
Father to Father	PASU _Father_	Frequency _Father_	0.06 **	0.02	0.003
		Quality _Father_	0.10 ***	0.02	<0.001
		Hands-on _Father_	−0.03	0.02	0.101
		Sequence _Father_	0.00	0.03	0.940
		Acquaintance _Father_	0.03	0.03	0.343
		Autonomy _Father_	0.36 ***	0.03	<0.001
		Co-use _Father_	0.27 ***	0.02	<0.001
		Knowledge _Father_	0.07 **	0.02	0.001
Father to Mother	PASU _Father_	Frequency _Mother_	−0.03	0.02	0.206
		Quality _Mother_	−0.05 *	0.02	0.028
		Hands-on _Mother_	−0.02	0.02	0.345
		Sequence _Mother_	−0.05	0.03	0.085
		Acquaintance _Mother_	−0.02	0.03	0.441
		Autonomy _Mother_	−0.02	0.03	0.442
		Co-use _Mother_	0.03	0.02	0.187
		Knowledge _Mother_	−0.03	0.03	0.276
Mother to Father	PASU _Mother_	Frequency _Father_	−0.03	0.02	0.206
		Quality _Father_	0.04	0.02	0.088
		Hands-on _Father_	0.04	0.02	0.080
		Sequence _Father_	−0.05	0.03	0.085
		Acquaintance _Father_	−0.02	0.03	0.441
		Autonomy _Father_	−0.02	0.03	0.442
		Co-use _Father_	0.03	0.02	0.187
		Knowledge _Father_	0.06 *	0.03	0.020
Mother to Mother	PASU _Mother_	Frequency _Mother_	0.06 **	0.02	0.003
		Quality _Mother_	0.10 ***	0.02	<0.001
		Hands-on _Mother_	−0.03	0.02	0.101
		Sequence _Mother_	0.00	0.03	0.940
		Acquaintance _Mother_	0.03	0.03	0.343
		Autonomy _Mother_	0.36 ***	0.03	<0.001
		Co-use _Mother_	0.27 ***	0.02	<0.001
		Knowledge _Mother_	0.07 **	0.02	0.001
Parents to Child	Frequency _Father_	PMU	0.04	0.04	0.212
	Frequency _Mother_	PMU	0.04	0.04	0.212
	Quality _Father_	PMU	−0.14 **	0.04	0.001
	Quality _Mother_	PMU	−0.31 ***	0.04	<0.001
	Hands-on _Father_	PMU	−0.02	0.04	0.723
	Hands-on _Mother_	PMU	−0.21 ***	0.05	<0.001
	Sequence _Father_	PMU	0.01	0.02	0.723
	Sequence _Mother_	PMU	0.01	0.02	0.723
	Acquaintance _Father_	PMU	0.01	0.01	0.620
	Acquaintance _Mother_	PMU	0.01	0.01	0.620
	Autonomy _Father_	PMU	0.04	0.03	0.088
	Autonomy _Mother_	PMU	0.04	0.03	0.088
	Co-use _Father_	PMU	0.01	0.06	0.858
	Co-use _Mother_	PMU	0.01	0.06	0.858
	Knowledge _Father_	PMU	−0.06	0.04	0.087
	Knowledge _Mother_	PMU	−0.06	0.04	0.087
Direct effects	PASU _Father_	PMU	0.01	0.03	0.836
	PASU _Mother_	PMU	0.24 ***	0.03	<0.001
	Child age	PMU	0.03	0.02	0.120
		Frequency _Father_	0.01	0.02	0.685
		Quality _Father_	0.04 *	0.02	0.022
		Hands-on _Father_	−0.02	0.02	0.292
		Sequence _Father_	−0.01	0.03	0.809
		Acquaintance _Father_	−0.03	0.03	0.256
		Autonomy _Father_	0.07 *	0.03	0.019
		Co-use _Father_	0.00	0.02	0.889
		Knowledge _Father_	−0.03	0.02	0.164
		Frequency _Mother_	0.01	0.02	0.540
		Quality _Mother_	0.02	0.02	0.163
		Hands-on _Mother_	−0.02	0.02	0.310
		Sequence _Mother_	0.01	0.03	0.622
		Acquaintance _Mother_	−0.05	0.03	0.085
		Autonomy _Mother_	0.06	0.03	0.075
		Co-use _Mother_	−0.04	0.02	0.107
		Knowledge _Mother_	−0.05 *	0.02	0.011
	Child gender	PMU	−0.06	0.03	0.099
		Frequency _Father_	−0.05	0.03	0.114
		Quality _Father_	−0.02	0.03	0.461
		Hands-on _Father_	−0.02	0.03	0.443
		Sequence _Father_	0.00	0.05	0.987
		Acquaintance _Father_	0.01	0.04	0.890
		Autonomy _Father_	−0.04	0.05	0.415
		Co-use _Father_	0.02	0.03	0.490
		Knowledge _Father_	−0.03	0.03	0.288
		Frequency _Mother_	0.01	0.03	0.784
		Quality _Mother_	0.00	0.03	0.904
		Hands-on _Mother_	0.00	0.03	0.952
		Sequence _Mother_	−0.10 *	0.05	0.029
		Acquaintance _Mother_	−0.04	0.05	0.345
		Autonomy _Mother_	−0.01	0.05	0.863
		Co-use _Mother_	−0.06	0.04	0.071
		Knowledge _Mother_	−0.08 *	0.03	0.010
	Num. of Children	PMU	0.00	0.03	0.951
		Frequency _Father_	−0.10 **	0.03	0.001
		Quality _Father_	−0.07 **	0.02	0.007
		Hands-on _Father_	−0.07 **	0.02	0.003
		Sequence _Father_	0.03	0.04	0.518
		Acquaintance _Father_	−0.02	0.04	0.539
		Autonomy _Father_	0.03	0.04	0.529
		Co-use _Father_	−0.10 **	0.03	0.003
		Knowledge _Father_	−0.10 **	0.03	0.001
		Frequency _Mother_	−0.11 ***	0.03	<0.001
		Quality _Mother_	−0.03	0.03	0.182
		Hands-on _Mother_	−0.06 *	0.02	0.011
		Sequence _Mother_	0.12 **	0.04	0.004
		Acquaintance _Mother_	0.08	0.04	0.068
		Autonomy _Mother_	0.01	0.05	0.825
		Co-use _Mother_	−0.05	0.03	0.099
		Knowledge _Mother_	−0.05	0.03	0.056
	SES	PMU	0.01	0.02	0.368
		Frequency _Father_	0.01	0.02	0.422
		Quality _Father_	0.01	0.01	0.492
		Hands-on _Father_	0.01	0.01	0.442
		Sequence _Father_	−0.04	0.02	0.086
		Acquaintance _Father_	−0.10 ***	0.02	<0.001
		Autonomy _Father_	−0.06 *	0.02	0.013
		Co-use _Father_	−0.01	0.02	0.615
		Knowledge _Father_	0.02	0.01	0.124
		Frequency _Mother_	0.09 ***	0.02	<0.001
		Quality _Mother_	0.07 ***	0.01	<0.001
		Hands-on _Mother_	0.06 ***	0.01	<0.001
		Sequence _Mother_	0.00	0.02	0.822
		Acquaintance _Mother_	−0.06 **	0.02	0.005
		Autonomy _Mother_	−0.04	0.03	0.166
		Co-use _Mother_	0.01	0.02	0.497
		Knowledge _Mother_	0.08 ***	0.01	<0.001

Note: * *p* < 0.05, ** *p* < 0.01, *** *p* < 0.001.
